# Assessing the effectiveness of the patient referral system in remote Zambia: A case study of Mbala District

**DOI:** 10.4102/phcfm.v18i1.5237

**Published:** 2026-08-29

**Authors:** Austine Dolopo

**Affiliations:** 1 Department of Graduate Studies, School of Business, University of Zambia, Lusaka, Zambia; 2 Department of Business Studies, School of Law & Business Studies, Eden University, Lusaka, Zambia

**Keywords:** patient referral system, emergency healthcare, health service accessibility, primary health care, rural healthcare, health systems

## Abstract

**Background:**

Effective patient referral systems are vital for ensuring timely access to advanced healthcare services, particularly in remote areas. In Zambia’s rural districts such as Mbala, the referral system faces critical challenges, including poor road networks, limited ambulance services, unreliable communication infrastructure and a lack of medical personnel at lower-level facilities. These issues contribute to delays, preventable complications, and increased patient morbidity and mortality.

**Aim:**

This study aims to assess the effectiveness of the patient referral system in Mbala District by examining logistical, infrastructural and human resource constraints that hinder timely and appropriate patient transfers from rural health facilities to higher-level care.

**Setting:**

The study was conducted in Mbala District, a predominantly rural district in Zambia’s Northern province, where geographical barriers, limited health infrastructure, and resource constraints present significant challenges to the effectiveness of the patient referral system.

**Methods:**

This study employed a descriptive cross-sectional design. Data were collected over 2 weeks using questionnaires from 18 health facility in-charges and community interviews, then analysed using Jeffreys’s Amazing Statistics Program (JASP) version 0.19.3 to evaluate referral accessibility, timeliness and effectiveness.

**Results:**

Statistical analyses show that communication reliability is the strongest predictor of referral success and satisfaction (*β* = 0.63, *p* = 0.002), also reducing delays by 1.89 h (*β* = –1.89, *p* = 0.007). Distance negatively affects referrals (*r* = –0.52), with over 70% of facilities beyond 25 km. Grid-powered facilities face 16-h daily power outages. A chi-square test found no significant link between community awareness and satisfaction (*χ*^2^ = 1.68, *p* = 0.1951).

**Conclusion:**

The referral system in Mbala District is significantly constrained by logistical and structural barriers. Targeted interventions, including improved transport logistics, communication infrastructure and staff training, are necessary to enhance patient outcomes in rural settings.

**Contribution:**

This study contributes empirical evidence on referral barriers in rural Zambia, highlighting communication, transport, distance, infrastructure and staffing as critical determinants of timely and effective patient referrals.

## Introduction

A well-functioning patient referral system is a critical component of an effective healthcare delivery model, particularly in rural and remote settings.^1^ It ensures that patients are transferred from primary care facilities to higher-level centres in a timely and organised manner, especially when specialised treatment is required.^2^ In Zambia, where health services are delivered through a three-tiered system, comprising primary, secondary and tertiary levels an efficient referral mechanism is essential for linking patients to appropriate levels of care.^3^ However, in remote areas such as Mbala District in Northern province, the referral system faces substantial challenges that undermine the delivery of equitable and quality healthcare. Globally, the importance of efficient referral systems is recognised under Sustainable Development Goal 3 (SDG 3), which seeks to ‘ensure healthy lives and promote well-being for all at all ages’. One of its key targets is to achieve universal health coverage (UHC), including access to quality essential healthcare services and timely referral interventions.^4^ Universal health coverage cannot be attained if patients in rural areas are unable to access higher levels of care because of transport barriers, weak infrastructure or a lack of emergency response mechanisms.^5,6^ Therefore, strengthening the referral system is central to fulfilling this global health mandate. In the context of Zambia, the National Health Strategic Plan (NHSP) 2022–2026, extended in subsequent frameworks, outlines the government’s commitment to ensure that all Zambians, particularly those in rural and underserved areas, have access to essential health services.^7^ The plan emphasises the importance of improving referral systems by addressing gaps in ambulance services, transport logistics, communication and staffing. Despite these policy aspirations, rural areas such as Mbala continue to face major limitations in implementing an effective referral chain. These challenges result in delays or failures in transferring patients to higher levels of care, ultimately compromising health outcomes. Emergency cases that require urgent attention such as maternal complications, severe injuries or paediatric emergencies, often face life-threatening delays.^8^ The inefficiencies in the referral system not only place additional strain on one general hospital available (servicing Mpulungu, Senga and Mbala District and also neighbouring Tanzania boarder areas) but also erode public confidence in the health system.^9^ Therefore, assessing the effectiveness of the patient referral system in Mbala District is both timely and necessary. This study aims to generate evidence that can inform policy adjustments and practical interventions aligned with SDG 3, Zambia’s health strategies and the broader goal of UHC. By identifying the structural, logistical and human resource barriers within the current system, this research will contribute to the development of a more equitable, responsive and resilient healthcare delivery system in rural Zambia.

## Research methods and design

### Study design

The design employed was selected to enable a systematic assessment of the effectiveness of the patient referral system across various health facilities and communities within Mbala District at a single point in time.

### Study setting

The study was conducted in Mbala District, one of the more remote districts in Zambia, located in the Northern province. The district has a total population of approximately 166 766 and is largely rural, characterised by difficult terrain, poor road networks and limited access to higher-level health services. Mbala has 29 health facilities, including rural health centres, health posts and Mbala General Hospital which serves as the referral centre for the area.

### Population

The study population comprised two main groups: (1) health facility in-charges responsible for coordinating patient referrals and (2) community members from villages served by the sampled health facilities. The inclusion of community members aimed to gather first-hand perspectives on their experiences with the referral system.

### Sample and sampling

Out of the 29 health facilities in the district, 18 were purposively sampled to ensure a representative mix of both hard-to-reach and more accessible facilities. At each of these facilities, the health facility in-charge was selected as a respondent. In addition, in every village visited during the study, 10 community members were interviewed, resulting in a total of 180 community respondents. Community members were selected using simple random sampling from households within the catchment area of each sampled facility. Data collection was completed within a 2-week period through a well-structured and coordinated fieldwork plan. A team of nine trained field assistants were deployed, organised into three teams of three members each, with each team responsible for covering six health facilities, ensuring efficient coverage of all sampled sites.

### Data collection

Data were collected using two tools: structured questionnaires administered to in-charges of 18 sampled health facilities, and community interview schedules conducted with 10 residents in each village visited. The questionnaires explored referral transport, communication systems, emergency response and staffing, while community interviews focused on accessibility, timeliness and satisfaction with referrals. Data collection spanned 2 weeks and was conducted by trained field assistants. Ethical standards, including informed consent and confidentiality, were strictly observed. Key variables included referral transport availability, communication systems, emergency response capacity, staffing levels, referral timeliness, accessibility of services and client satisfaction. These variables were used to assess the efficiency and effectiveness of the patient referral system. The data were coded and analysed using Jeffreys’s Amazing Statistics Program (JASP) version 0.19.3 statistical software to generate descriptive statistics and identify trends in the effectiveness of the referral system across Mbala District.

### Ethical considerations

Ethical clearance to conduct this study was obtained from the Eden University School of Medicine Research Ethics Committee (No. REF. No. EdenUSMC-01-06-2025).

## Results

Table 1 and Table 2 reveal significant challenges related to distance, load-shedding, and other infrastructural constraints within the district. Nine health facilities are located more than 26 km from the General Hospital, while seven are situated beyond 50 km. These considerable distances are compounded by poor road infrastructure, which becomes particularly difficult to navigate and, in some cases, impassable during the rainy season, thereby contributing to delays in patient transfers.

**TABLE 1:** Systemic barriers affecting the referral system in Mbala District (*N* = 18).

Variable	District status
Limited ambulance availability	One operational ambulance serving the district
Poor road infrastructure	Roads frequently impassable during rainy season
Unreliable communication networks	Mobile network coverage unreliable in several areas
Absence of resident medical doctors	No resident medical doctors in rural facilities
Unreliable power supply	Frequent electricity interruptions reported

*Source:* Ministry of Health. Northern Province: Mbala District Health Office Medium Term Expenditure Framework (MTEF) Action Plan for 2024 to 2026^10^

Note: Mean prevalence (%) = 100.

**TABLE 2:** Distances of health facilities from the general hospital in Mbala District, Zambia (2025), *N* = 18.

Distance category (km)	Number of facilities
0 km – 25 km	3
26 km – 50 km	8
51 km – 75 km	7
76 km – 100 km	1

*Source:* Ministry of Health. Northern Province: Mbala District Health Office Medium Term Expenditure Framework (MTEF) Action Plan for 2024 to 2026^10^

Furthermore, unreliable power supply and prolonged load-shedding, averaging 16 h per day, significantly constrain the capacity of rural health facilities to stabilise and appropriately manage patients before referral. This further compromises the timeliness, continuity, and effectiveness of the referral process.

The situation is exacerbated by the absence of resident medical doctors in rural health facilities, limiting their capacity to manage critical and complicated cases at the point of care and increasing reliance on an already strained referral system. In addition, unreliable mobile communication networks impede timely coordination between referring and receiving facilities, particularly during emergencies, thereby increasing the risk of delays and potentially adverse patient outcomes.

Table 3 highlights the energy access constraints among grid-powered health facilities in Mbala District. All four facilities, Zombe Rural Health Post, Kamuzwazi Rural Health Centre, Kawimbe Rural Health Centre and Mwamba Rural Health Centre experience an average of 16 h of loadshedding per day, regardless of their proximity to the district hospital, which ranges from 18 km to 45 km.

**TABLE 3:** Loadshedding hours and distance among grid-powered health facilities in Mbala District – Zambia (2025, *N* = 4).

Facility name	Distance to hospital (km)
Zombe RHP	18
Kamuzwazi RHC	45
Kawimbe RHC	23
Mwamba RHC	37
Number of assessed facilities	4

*Source:* Ministry of Health. Northern Province: Mbala District Health Office Medium Term Expenditure Framework (MTEF) Action Plan for 2024 to 2026^10^

Note: Mean daily loadshedding hours = 16 h/day.

RHC, rural health centre; RHP, rural health post.

One of the key findings is a moderate negative correlation between the distance to the hospital and referral success (*r* = –0.52). A strong positive correlation (*r* = 0.64) was found between staff preparedness and referral success. Ambulance availability also demonstrated a moderate positive correlation (*r* = 0.55) with referral success. The most pronounced finding from the analysis is the very strong positive correlation (*r* = 0.89) between mobile network coverage and referral success. Improving network coverage in rural areas is a priority intervention for improving health system responsiveness and patient outcomes.

### Findings of regression results

Regression analysis revealed that road condition, transport availability, communication reliability and facility type were important factors in the referral system performance across health facilities in Mbala District. The results showed varying effects of these factors on both referral delay and referral satisfaction. Facilities with better road access, more reliable transport and dependable communication systems generally experienced shorter referral delays and reported higher levels of satisfaction with the referral process. Differences were also observed across facility types, highlighting the influence of infrastructure and operational capacity on the efficiency and perceived quality of referral services.

### Referral delay model

In the referral delay model, the following coefficients were observed:

- road condition (β = –1.64, *p* = 0.017)
- communication reliability (β = –1.89, *p* = 0.007)
- transport availability (β = –0.97, *p* = 0.224)
- facility type (β = –0.55, *p* = 0.478)
- constant (β = 10.47, *p* = 0.001).

The negative and statistically significant coefficient for road conditions implies that as road conditions improve (from poor to good), referral delays decrease by approximately 1.64 h, all else held constant. Similarly, communication reliability shows a strong and significant effect: for every unit increase in communication reliability (e.g. from poor to moderate), referral delays reduce by nearly 1.89 h. Although transport availability and facility type showed negative coefficients suggesting that they help to reduce delays, their *p*-values (0.224 and 0.478, respectively) indicate that these associations were not statistically significant at the 5% level.

### Referral satisfaction model

In the referral satisfaction model, the coefficients were:

- communication reliability (β = +0.63, *p* = 0.002)
- transport availability (β = +0.27, *p* = 0.176)
- facility type (β = +0.21, *p* = 0.342)
- road condition (β = +0.19, *p* = 0.234)
- constant (β = 2.01, *p* = 0.002).

Here, communication reliability again stood out with a statistically significant positive effect. A one-point improvement in communication reliability is associated with a 0.63-point increase in satisfaction, on a 5-point scale.

## Discussion

### Systemic barriers affecting the referral system in Mbala District

The results in Mbala District highlight several systemic weaknesses: the presence of only one operational ambulance for the entire district; poor road infrastructure that becomes impassable during the rainy season; and unreliable mobile communication networks that impede timely coordination during emergencies. Compounding these logistical barriers is the absence of resident medical doctors in rural health facilities, meaning that critical cases cannot be managed locally and must rely on the already-strained referral pathway. Unreliable sources of power in rural facilities further limit their capacity to stabilise patients before transfer.

### Distribution of health facilities by distance

The distribution of health facilities by distance from the general hospital offers critical insights into the structural limitations of the referral system in Mbala District. The spatial distribution poses significant challenges for timely patient referrals, especially in emergency situations. Evidence from rural health systems in sub-Saharan Africa consistently indicates that greater distances from referral centres are associated with increased mortality and delayed care, particularly in obstetric and paediatric emergencies.^11^ Kruk et al. argue that physical access is among the most persistent barriers to achieving equitable health outcomes in low-resource settings.^12^ In Zambia, the Ministry of Health recognises distance as a major determinant of care-seeking delays, as highlighted in the Zambia Demographic and Health Survey (ZDHS).^13^ Poor road networks, limited transport options and high fuel costs further compound the issue.^14^ Furthermore, long distances have been shown to reduce the frequency of completed referrals, as patients may abandon transfer because of travel difficulty or cost.^15^ In Mbala’s context, these constraints are intensified by the presence of only one district ambulance and poor communication network. To address this, targeted interventions such as investment in community-based transport solutions and improved road infrastructure are essential. Equally important is integrating telehealth and mobile-based coordination to bridge the gap for distant communities.^16^

### The role of electricity in the health system

The level of power outage in Mbala District represents a critical operational challenge, particularly for managing emergency referrals that require refrigeration of drugs, lighting during night-time emergencies and the use of diagnostic equipment. In rural health systems, reliable electricity is essential for effective service delivery. Power outages are directly linked to reduced healthcare quality and increased maternal and neonatal risks.^17^ The World Health Organization (WHO) also emphasises the role of electricity in supporting functioning cold chains, communication systems and life-saving interventions. Mbala’s grid-connected facilities, while technically electrified, remain functionally underserved because of prolonged power outages, with only a few facilities supplementing grid electricity through solar energy systems.^8^ Moreover, loadshedding disproportionately impacts referral systems, as communication tools (e.g. phones, radios) often depend on electricity. As observed by Scott et al., electricity access significantly enhances the operational readiness of rural health facilities.^18^ Without reliable power, even facilities near the referral hospital, such as Zombe (18 km), remain handicapped in managing emergency care. Therefore, investing in alternative sources such as solar systems with battery storage is vital. This aligns with recommendations by the Sustainable Energy for All initiative (SEforALL), which promotes decentralised energy solutions for rural health infrastructure.^19^

### Correlation analysis: Bivariate of referral success in Mbala District

Various studies conducted in rural and resource-constrained settings, particularly in sub-Saharan Africa, show that referral systems are influenced by multiple factors, including infrastructure, transport, communication and human resource capacity. These factors highlight the need for integrated, evidence-based strategies to strengthen referral systems across remote districts, as discussed in the subsequent sections.^20^ As shown in Table 4, a moderate negative correlation between distance to the hospital and referral success (*r* = –0.52) reflects barriers associated with geographic isolation. Numerous studies confirm that long distances to referral centres significantly delay or prevent access to advanced care. Kruk emphasises that geographical barriers are among the most persistent determinants of poor health outcomes in rural areas, highlighting the negative consequences of delayed access to emergency services because of distance.^21^ In Zambia, the Ministry of Health, in collaboration with development partners, has consistently documented through the ZDHS that women in remote areas are less likely to access timely obstetric and emergency care. A strong positive correlation between staff preparedness and referral success (*r* = 0.64) indicates that frontline health worker competence, including knowledge of referral protocols, triage ability and emergency stabilisation, plays a pivotal role in saving lives. Waiswa’s research team found that in rural Uganda, maternal referral outcomes were significantly influenced by the responsiveness and preparedness of health personnel.^22^ Studies have highlighted the essential role of emergency transport in effective health system functioning. Awoonor-Williams et al. demonstrated in Ghana that increased availability and reliability of ambulances improved patient transfer rates and outcomes in rural districts.^23^ Likewise, Kipuro et al., indicated that districts equipped with functioning ambulances report higher completion rates of referral cases, although this is conditional on the availability of fuel, trained drivers and operational support.^24^ Network coverage exhibited the strongest positive correlation with referral success (*r* = 0.89), emphasising the indispensable role of communication. Studies have consistently identified reliable communication as a critical enabler of timely referrals. Atukunda et al. reported that rural health workers in Uganda using mobile phones were able to reduce delays and coordinate emergency responses more efficiently.^25^ Similarly, WHO and United Nations Children’s Fund (UNICEF) have underscored that robust communication systems are vital for emergency preparedness, especially in underserved and remote areas.^26^

**TABLE 4:** Correlation analysis: Bivariate of referral success in Mbala District – Zambia (2025, *N* = 18).

Variable	Correlation with referral success
Distance to hospital	−0.52
Staff preparedness	0.64
Ambulance availability	0.55
Network coverage	0.89

*Source:* Ministry of Health. Northern Province: Mbala District Health Office Medium Term Expenditure Framework (MTEF) Action Plan for 2024 to 2026^10^

### Referral delay and satisfaction

The referral satisfaction model provides key insights into the determinants of user satisfaction with the patient referral system in Mbala District. Among the predictors evaluated, communication reliability emerged as the only statistically significant factor (β = 0.63, *p* = 0.002), indicating a strong and positive relationship with satisfaction. A one-point increase in the communication reliability score corresponds to a 0.63-point rise in satisfaction on a 5-point scale. This finding is consistent with evidence suggesting that timely, reliable communication enhances user confidence in referral systems and reduces frustration associated with delays and uncertainty.^27^ In contrast, transport availability (β = 0.27, *p* = 0.176), road condition (β = 0.19, *p* = 0.234) and facility type (β = 0.21, *p* = 0.342) were positively associated with satisfaction but did not achieve statistical significance. These results suggest that while physical infrastructure and institutional capacity are important, they are secondary to the perceived effectiveness of communication channels in shaping satisfaction. As highlighted by Peters et al., patient satisfaction in low-resource settings is often driven by system responsiveness and accessibility rather than facility classification.^20^ This aligns with research by Ouma et al., who found that weak communication mechanisms are often more limiting than geographic distance in the success of rural health interventions.^21^ Similarly, Kruk et al. emphasise that functional systems where patients are guided, informed and reassured foster trust and satisfaction, regardless of structural limitations.^12^ Moreover, WHO advocates for integrated digital health tools to facilitate real-time coordination and feedback between service points.^26^ Therefore, enhancing communication infrastructure and protocols in rural Zambia could serve as a high-impact intervention for improving referral satisfaction, even in the absence of immediate improvements in roads or transport.

### Community awareness and satisfaction

The cross-tabulation presented in Table 5 explores the relationship between community awareness of the patient referral system and their reported satisfaction with the referral services in Mbala District. Among the respondents, a higher proportion of those who were aware of the referral system reported not satisfied compared to those who were not aware. The results suggest that while awareness may influence satisfaction at a superficial level, it is not, in itself, a strong determinant of how community members perceive the referral system. These findings underscore that satisfaction with healthcare services, particularly in rural and low-resource settings, is often influenced by a constellation of factors beyond mere awareness. According to Peters et al., physical accessibility, financial barriers, quality of care and responsiveness of healthcare workers play a more decisive role in shaping user experiences.^20^ Furthermore, Atkinson et al., emphasise that in health systems where logistical challenges such as transport, delays and poor infrastructure persist, awareness without corresponding system performance improvements does little to improve satisfaction.^15^ Nonetheless, community awareness remains a foundational component of health system engagement.^26^ It enhances health-seeking behaviour and enables community members to better understand their rights and the services available to them. Therefore, while awareness alone may not guarantee satisfaction, it is a prerequisite for facilitating timely and appropriate referrals. Efforts to enhance satisfaction should therefore adopt a holistic approach combining awareness campaigns with improvements in emergency transport, infrastructure, health worker capacity and communication systems.

**TABLE 5:** Cross-tabulation of community awareness of the referral system and reported satisfaction levels among respondents in Mbala District (2025, *N* = 60).

Awareness	Not satisfied	Satisfied
No	9	16
Yes	26	9

*Source:* Ministry of Health. Northern Province: Mbala District Health Office Medium Term Expenditure Framework (MTEF) Action Plan for 2024 to 2026^10^

Note: Chi-square statistic (*χ*^2^) = 0.33. Degrees of freedom = 1. *p* = 0.5677. The test yielded a chi-square value of 0.33 with 1 degrees of freedom and a *p*-value of 0.5677.

## Conclusion

This study assessed the effectiveness of the patient referral system in Mbala District, focusing on logistical, infrastructural and systemic factors influencing referral delays and satisfaction. The findings reveal significant structural weaknesses that compromise the efficiency and reliability of the referral system in rural health settings. Statistical analyses indicate that communication reliability is the most influential determinant of referral success and satisfaction. Regression results show a strong positive association between communication reliability and referral satisfaction (β = 0.63, *p* = 0.002), highlighting the critical role of real-time coordination in emergency response. In addition, improved communication was associated with a reduction in referral delays by 1.89 h on average (*β* = –1.89, *p* = 0.007). Distance to the general hospital exhibited a moderate negative correlation with referral success (*r* = –0.52), affirming that facilities located beyond 25 km comprising over 70% of health centres in the district face greater challenges in executing timely referrals. Loadshedding data further revealed that all grid-connected facilities experience 16 h of power outage per day, severely limiting operational capacity. The chi-square test showed no statistically significant association between community awareness and referral satisfaction (*χ*^2^ = 1.68, *p* = 0.1951), suggesting that awareness must be accompanied by system performance to meaningfully influence satisfaction levels. Strengthening communication infrastructure, alongside targeted improvements in road access, energy reliability and staff preparedness, is essential for improving referral outcomes in Mbala District. The evidence supports the integration of mobile-based referral coordination tools, investment in credible solar energy systems and decentralisation of referral services to reduce delays and improve patient care. Addressing these determinants holistically can significantly enhance the responsiveness, equity and overall performance of rural referral systems in Zambia and similar contexts.

### Limitations of the study

This study is limited by a small sample size, which may reduce statistical power and obscure significant relationships. Furthermore, reliance on self-reported data introduces potential bias, and the cross-sectional design prevents causal inferences. Unmeasured variables, such as fuel availability and staff workload, may also influence referral outcomes. The use of only one respondent per facility may have introduced response biasness.
